# Materials-based incidence of urinary catheter associated urinary tract infections and the causative micro-organisms: systematic review and meta-analysis

**DOI:** 10.1186/s12894-024-01565-x

**Published:** 2024-08-30

**Authors:** Benjamin Gambrill, Fabrizio Pertusati, Stephen Fon Hughes, Iqbal Shergill, Polina Prokopovich

**Affiliations:** 1 School of Pharmacy and Pharmaceutical Sciences, Cardiff University, King Edward VII Avenue, Cardiff, CF10 3NB UK; 2 Maelor Academic Unit of Medical Surgical Sciences (MAUMSS), Betsi Cadwaladr University Health Board (BCUHB), Wrexham, North Wales; 3 The Alan de Bolla Department of Urology, BCUHB Wrexham Maelor Hospital, Wrexham, North Wales

**Keywords:** Antibiotic resistance, Urinary catheter, UTI, CAUTI, Nosocomial infection, Silver-alloy, Urology

## Abstract

**Background:**

Both long (> 30 days) and short-term (≤ 30 days) catheterisation has been associated with urinary tract infections (UTIs) due to the invasive nature of device insertion through the urethra. Catheter associated Urinary Tract Infections (CAUTIs) are common (prevalence of ~ 8.5%) infections which can be treated with antibiotics; however, CAUTIs are both expensive to treat and contributes to the antibiotic usage crisis. As catheters are unlikely be replaced for the management of patients’ urination, ways of reducing CAUTIs are sought out, using the catheter device itself. The aim of this review is to assess the incidence of CAUTI and the causative micro-organisms when different urinary catheter devices have been used by humans, as reported in published research articles.

**Methods:**

A Systematic Literature Review was conducted in Ovid Medline, Web of Science and PubMed, to identify studies which investigated the incidence of UTI and the causative micro-organisms, in patients with different urinary catheter devices. The articles were selected based on a strict set of inclusion and exclusion criteria. The data regarding UTI incidence was extracted and calculated odds ratio were compared across studies and pooled when types of catheters were compared. CAUTI causative micro-organisms, if stated within the research pieces, were also gathered.

**Results:**

A total of 890 articles were identified, but only 26 unique articles met the inclusion/exclusion criteria for this review. Amongst the large cohort there were catheters of materials silicone, latex and PVC and catheter modifications of silver nanoparticles and nitrofurantoin antibiotics. The meta-analysis did not provide a clear choice towards a single catheter against another although silver-based catheters, and silver alloy, appeared to statistically reduce the OR of developing CAUTIs. At genus level the three commonest bacteria identified across the cohort were *E. coli*, *Enterococcus spp.* and *Pseudomonas spp.* whilst considering only at the genus level, with *E. coli*,* Klebsiella pneumonia* and *Enterococcus faecalis* most common at the species-specific level.

**Conclusions:**

There does not appear to be a catheter type, which can significantly reduce the incidence of CAUTI’s in patients requiring catheterisation. Ultimately, this warrants further research to identify and develop a catheter device material that will reduce the incidence for CAUTIs.

**Supplementary Information:**

The online version contains supplementary material available at 10.1186/s12894-024-01565-x.

## Introduction

UTIs are estimated to represent up to 40% of all nosocomial infections [1–3]. UTIs arising in hospital environments include catheter associated urinary tract infections (CAUTI). Approximately 20% of hospital-acquired bacteraemia arise from the CAUTI [4] with an incidence of 3–8% per day [5–9] and a prevalence of about 8.5% [10]. Beside patient discomfort and economic impact on the health provider, mortality associated to UTI-associated bacteraemia is approximately 10% [11]. Despite these numbers, catheters are still needed by patients with acute urinary retention or bladder outlet obstruction [12]. In a cohort of 931,945 adults receiving primary care with ages ≥ 65 years at least one UTI was diagnosed in 21% of the cohort, demonstrating a considerable UTI incidence [13]. Both long (≥ 30 days) [14] and short-term (≤ 30 days) [15] catheterisation can result in UTI; however, this more likely for long-term catheter usage [16, 17]. Despite the duration of short-term catheterisation, it was found to have a 5% increase in the daily infection rate [5, 18]. Use of indwelling catheters requires passing through the urinary tract via urethra to reach the bladder, where the catheter is held place by balloon inflation. This invasive nature of indwelling catheterisation through the urinary canal for long durations possesses a greater risk of CAUTI than intermittent catheterisation, where the catheter is frequently cleaned or exchanged for a new catheter [19]. As such, use of urinary catheters is avoided if possible with intermittent catheterisation preferred [20]. However, the increase of risk using indwelling catheters over intermittent ones is debated [21]. Commercially available catheter devices can exist in a variety of different types and materials, all of which exhibit smooth outer surfaces for patient comfort and ease of passage through the urinary tract [22].

*Escherichia coli*,* Proteus spp.*,* Staphylococcus saprophyticus* and *Klebsiella spp*. are commonly the causative of micro-organisms in CAUTI [23]. Despite the prevalence of antibiotic resistance especially in hospital environments, antibiotics are still frequently used due to prescribing habits stemming from the ‘golden age of antibiotic discovery’ [23–26]. Deaths worldwide attributed to bacterial resistance to antibiotics was estimated at 4.95 million in 2019 and expected to rise to around 10 million in 2050 [27]. In England deaths attributable to antibiotic resistance in 2019 was 2596, a number which has increased since 2016 [28]. However, antibiotics are required to treat complicated UTIs, where specific considerations are made regarding drug selection [29]. Therefore, with the likelihood of both CAUTI and antibiotic resistance arising it is justified that significant work has been dedicated to novel prophylactics to tackle infection. With the ever-growing antibiotic resistance problem, non-antibiotic drug based approaches are being extensively studied in order to target bacteria in ways they do not possess resistance mechanisms against. Since use of catheters devices in healthcare cannot be avoided efforts have been made to incorporate prophylactics into the catheter devices themselves.

With an increased risk of nosocomial infections from patients using catheters, antimicrobials are often administered regardless the presence of infection [30]. CAUTIs become difficult to treat due to the formation of bacterial biofilm which increases bacterial persistence and resistance to antibiotics. Biofilm formation relies on bacterial factor Fimbriae type 1 (FimA) and PapC which are responsible for epithelial cell adhesion and pili attachment to urinary tract cells or catheter materials respectively [31]. Catheter use for seven days resulted in biofilm formation in up to 50% of cases whilst users of more than 28 days always experienced biofilm formation [32]. Urease activity by bacteria such as *Proteus mirabilis* results in increased environment pH from production of ammonia and carbon dioxide, which in turn results in crystallisation by calcium precipitation. Crystallization can occur within catheter lumen and upon urinary tract wall which can block drainage and cause patient discomfort respectively [33, 34]. Latex catheters were deemed to be most encouraging for bacterial biofilm formation [35]. As such, research is required to further identify the most suitable catheter material which considers both patient comfort and risk of UTI, including modifications to the material to incorporate antibacterial substances into the material itself which actively discourage bacterial infection. Despite the numerous studies using novel materials, these studies test the materials against bacterial cultures without being tested as whole catheters by human participants. As such, it is important to establish which modified materials have been tested in humans as whole catheter devices.

The aim of this research was to investigate the incidence of UTI when at least 2 different catheters are used. Specifically, this literature review provides an insight into catheter devices materials and modifications, which should be used to reduce the likelihood of UTI caused by the well reported micro-organisms. Ultimately, this review intends to aid and support clinicians and healthcare providers, with insights into catheter device performance and the micro-organisms which cause UTIs.

## Materials and methods and data source search strategy

Databases containing peer reviewed articles were searched (April 2022) using devised multiple string searches appropriate to the research questions. Searches were conducted in Ovid MEDLINE (Supplementary Table 1 A), PubMed (Supplementary Table 1B), and Web of Science (Supplementary Table 1 C).

### Eligibility criteria

Two authors independently evaluated research based on article title and abstract in line with set out inclusion/exclusion criteria. Further criteria consisted of clinical trial research published in the period between 2000 and 2022 and entire article published in English to provide relevancy and ease of understanding respectively. From the identified articles the title, author and abstract were extracted into Excel. To ensure relevancy to the research questions only studies where the catheter device used is responsible for the outcome of interest (Table 1) were selected. The remaining articles were fully read for a final assessment before being included in the final review.

**Table 1:** Inclusion and exclusion criteria

PICOS/Criteria	Inclusion	Exclusion
Population	Human ≥ 18 years old	Humans < 18 years old, studies on animals and cells
Intervention	Populations undergoing catheterisation	Populations NOT undergoing catheterisation
Outcome	Incidence of UTI or associated infections and causative micro-organisms	Studies which do not report incidence of UTI or associated infections e.g., only cost benefit analysis
Study type	Prospective or retrospective studies, Randomised or crossover studies	Case reports, commentary, letters, reviews
Language	Full study available in English	Studies in language other than English

### Data extraction

General information extracted from each study included such as reason for catheterisation and country in which the study was conducted. Specific information was also extracted: included number of participants, number of infections detected, duration of study and type of catheter used. If micro-organisms were identified their genus and species were also collected for comparison amongst the cohort.

### Quality assessment of included studies

Each article identified as suitable for meta-analysis was critiqued using the ‘Critical Appraisal Skills Programme’ (CASP) to assess their reliability and suitability in meeting the research aims and objectives. The CASP checklist used was the CASP Cohort Study Checklist [36] and from the list questions with answer options ‘yes’, ‘can’t tell’ and ‘no’ were used to assess the collected cohort. Low scoring studies would have been excluded and those with clear confounding factors, such as all participants being diabetic would have resulted in article exclusion.

### Statistical analysis

Odds Ratio (OR) of developing UTI in the cohort exposed to the novel tested material against patients in the control group were calculated along the 95% Confidence Intervals (CIs) in each study from numerical data of number of catheter users and number of infections measured in each trial arm. ORs were pooled in a meta-analysis using the Mantel-Haenszel method for fixed-effect models and the DerSimonian-Laird estimator for random-effect models for the assessment of which catheter devices were associated with lowest risks for UTI. Both fixed-effects and random-effects models were used when calculating odds ratio due to the variance in the number of studies using different catheter type. Meta-analysis calculations and Forest plots were constructed using the forestplot package in R (version 4.2.2). Given the different types of catheters used in the included studies, they were stratified in studies which used silver-based catheters or non-silver-based materials against standard catheters.

## Results

### Search results

Searches in Web of Science, Ovid Medline and PubMed each yielded 800, 18 and 72 studies, respectively. From the initial database searches studies prior to year 2000 and those which were reviews or notes, for example, were removed using database filter options. Despite using the database filter features, articles which were reviews remained and then identified during the initial abstract/title-based exclusion. From the 890 studies identified in the databases, 77 were removed as they were not unique.

**Fig. 1:**
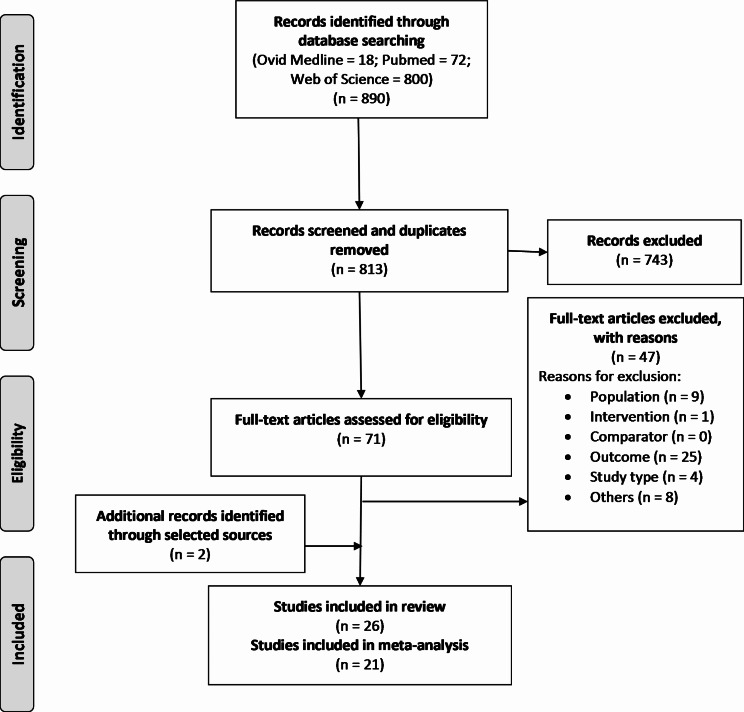
Preferred reporting items for systematic reviews and meta-analyses workflow for article identification

Article exclusion from title and abstract examination were for reasons such as animal usage (*n* = 38), in-vitro materials or bacterial culture usage (*n* = 146) or outcomes which lacked focus on catheter usage and UTI incidence (*n* = 458). As such, 743 articles were immediately excluded as they did not meet the inclusion criteria. Upon a readthrough of the remaining 70 articles the majority were discarded resulting in 24 articles. Reasons for exclusion were articles with only abstracts published in English (*n* = 4), articles where journal access was unavailable (*n* = 4) and review articles (*n* = 4). Population reasons for exclusion were those which had participants under the age of 18 (*n* = 5), those that used animals (*n* = 1) and research using materials instead of living organisms (*n* = 3). Most excluded articles were those that did not present UTI incidence and therefore excluded for outcome reasons. A further 2 articles were found whilst conducting thorough readthroughs which were suitable according to the inclusion criteria, totalling 26 articles for the narrative SLR. Of these 26 articles, 21 provided enough numerical data to calculate OR and 95% CI to conduct meta-analysis. Workflow of article exclusion was carried out using a Preferred Reporting Items for Systematic Reviews and Meta-analyses workflow (PRISMA) (Fig. 1).

### Cohort characteristics

Common characteristics were collected from the 26 identified articles (Table 2). Countries in which the studies were conducted in were ‘not reported’ (*n* = 6), USA (*n* = 5), UK (*n* = 3), Turkey (*n* = 2), Sweden (*n* = 2), Brazil (*n* = 2), Saudi Arabia (*n* = 1), India (*n* = 1), Italy (*n* = 1) and Hong Kong (*n* = 1). The remaining articles were studies conducted in more than one country (*n* = 2). The most frequent given reason for catheterisation were both spinal cord injuries (SCI) (*n* = 6) and non-neurological reasons (*n* = 6). Other reasons for catheterisation were ‘requiring patients’ (n = 7), other neurological conditions other than SCI (n = 4) and ‘not reported’ (n = 3). Some studies declared the number of males and females in their studies (n = 19) or just the number of participants (n = 7). There was a total of 42,665 participants across the studies and from those which stated number of males (n = 4,176) and females (n = 3,647) the gender ratio was 1.15. Articles identified were either prospective (n = 24), retrospective (n = 1) or a combination of both (n = 1). The studies designs were either randomised controlled trial (n = 18), crossover study (n = 7) or a combination of both (n = 1). The variety of different study types was justified based on participants having catheter devices either as the start of the intervention (randomised controlled trial) or having a new catheter after being cleared for having CAUTI (crossover). As such, CAUTI events were reported while participants had a urinary catheter device was unique to having a particular catheter in place regardless of study type.

In terms of blinding there were all possible degrees amongst the cohort of research papers. Articles were either double-blinded (*n* = 3), single-blinded (*n* = 6), open-label (*n* = 10) or not reported (*n* = 7). Studies which were single blinded involved only patients not knowing which catheter type they were given, with hospital staff knowing as they were administrating the catheters, suggesting a reason as to why there were few double-blind studies. One study states that blinding was not possible given the difference of appearance of the catheters [37], which given that catheters are required to be in sterile packaging would be difficult to blind for those providing healthcare.

The catheters used as standard catheters included silicone (*n* = 10), PVC (*n* = 4), latex (*n* = 2), siliconised latex (*n* = 2), polytetrafluoroethylene-latex (*n* = 1) and novel silver release latex (*n* = 1). The remaining standard catheters used in the research were not specified in their material or supplier (*n* = 4). The catheters which were tested for their ability to reduce the incidence of UTI were silver-alloy latex (*n* = 11), silver-alloy silicone (*n* = 6) nitrofurazone silicone (*n* = 3), polyvinyl- coated pyrrolidone polyolefin-based elastomer (*n* = 2), polyvinyl-pyrrolidone polyurethane (*n* = 1), drug coated PVC (*n* = 1), gel coated PVC (*n* = 1), siliconised latex (*n* = 1) and silver salts latex (*n* = 1). UTI in patients was identified as either UTI or symptomatic bacteriuria whilst a urinary catheter is being used, where in both cases bacterial concentration in urine is 10 [5] CFU/mL [38].

**Table 2:** Cohort characteristics

Author (Publication year)	Country	Study design (Trial type) and nature of blinding	Catheterisation Period (days)	Type of standard catheter	Standard catheters users (infections)	Type of test catheter	Test catheter users (infections)
Akcam et al. (2019)[39]	Turkey	Prospective (RCT) Double-blind	22	Silicone	26 (12)	SAS	28 (13)
Aljohi et al. (2016)[40]	Saudi Arabia	Prospective (RCT)Single-blind	3	Siliconised Latex	30 (10)	SAL	30 (1)
Banaszek et al. (2017)[41]	NR	Prospective (RCT)Non-blind	27	Latex	178 (100)	SAS	124 (28)
Bonfill et al. (2017)[42]	Italy, Portugal, Spain, Turkey	Prospective (RCT)Single-blind	28	Silicone/Latex	246 (19)	SAL	243 (18)
Cardenas et al. (2009)[43]	USA	Prospective (RCT)Non-blind	365	‘Standard’	23 (14)	PPPE	22 (12)
Cardenas et al. (2011)[44]	USA and Canada	Prospective (RCT)Non-blind	NR	PVC	69 (76)	PPP	45 (41)
Chung et al. (2017)[45]	Hong Kong	Prospective (RCT + CS)Non-blind	17*	Silicone	306 (70)	SAL	306 (28)
Cindolo et al. (2003)[46]	Italy	Prospective (RCT)Single-blind	NR	PVC	50 (35)	Drug-PVC	50 (17)
Gentry et al. (2005)[47]	UK	Prospective (CS)NR	10*	‘Standard’	55 (4)	SAL	78 (4)
Kai-Larsen et al. (2021)[48]	India	Prospective (RCT)Non-blind	11*	Latex	250 (53)	SAL	750 (52)
Karchmer et al. (2000)[49]	USA	Prospective (CS)Non-blind	NR	Siliconised Latex	27,878 (176)	SAL	27,878 (115)
Lederer et al. (2014)[50]	USA	Retrospective (CS) Non-blind	11*	Silicone/Latex	453 (NR)	SAL	400 (NR)
Lee et al. (2004)[51]	NR	Prospective (RCT)NR	NR	Silicone	85 (19)	NFS	92 (14)
Leuck et al. (2015)[37]	USA	Prospective (RCT)Non-blind	30	NSRL	49 (0)	SAS	46 (1)
Magnusson et al. (2019)[52]	NR	Prospective + Retrospective (CS)Non-blind	NR	Silicone	1 (NR)	SAS	1 (NR)
Menezes et al. (2018)[53]	Brazil	Prospective (RCTNR	5*	Silicone	88 (6)	NFS	88 (7)
Pickard et al. (2012)[54]	UK	Prospective (RCT)NR	2*	PTFE-Latex	2,144 (271)	NFS	2,153 (228)
Pickard et al. (2012)[54]	UK	Prospective (RCT)NR	2*	PTFE-Latex	2,144 (271)	SAL	2,097 (263)
Sarcia et al. (2010)[55]	Turkey	Prospective (RCT)Single-blind	NR	PVC	25 (5)	Gel-PVC	25 (1)
Seymour (2006)[56]	UK	Prospective (CS)NR	17*	‘Standard’	54 (2)	SAL	63 (6)
Srinivasan et al. (2006)[57]	USA	Prospective (CS)NR	4*	Silicone	1,871 (218)	SAS	1,165 (116)
Stenzelius et al. (2011)[58]	Sweden	Prospective (RCT)Single-blind	2*	Silicone	217 (12)	SAL	222 (3)
Stenzelius et al. (2016)[59]	Sweden	Prospective (CS)Double-blind	8.8 ± 11.1	Silicone	171 (39)	SAS	151 (39)
Thibon et al. (2000)[60]	NR	Prospective (RCT)Double-blind	5.8 ± 2.5	Silicone	109 (13)	SSL	90 (9)
Vapnek et al. (2003)[61]	NR	Prospective (RCT)NR	NR	PVC	28 (20)	PPPE	27 (21)
Verma et al. (2016)[62]	NR	Prospective (RCT)Non-blind	5	Silicone	50 (12)	Siliconised Latex	50 (22)
Zampieri et al. (2020)[63]	Brazil	Prospective (RCT)Single-blind	326	‘Standard’	55 (3)	SAL	48 (2)

(**NR**: Not reported, **RCT**: Randomised controlled trial, **CS**: Crossover study, **PVC**: polyvinyl chloride, **PTFE**: polytetrafluoroethylene, **NSRL**: novel silver release latex, **SSL**: Silver salts latex, **SAS**: silver-alloy silicone, **SAL**: silver-alloy latex, **PPPE**: polyvinyl-pyrrolidone polyolefin-based elastomer, **PPP**: polyvinyl-pyrrolidone polyurethane, **NFS**: nitrofurazone silicone)

* : Mean number of days catheterised as given in article, Pickard et al. (2012) has been split into two entries as a three-way comparison of catheter

### Critical appraisal skills programme (CASP)

All included research were assessed as medium (7) to high (19) based on topic coverage using Critical Appraisal Skills Programme (CASP). CASP questions were answered with ‘yes’, ‘can’t tell’ or ‘no’ responses based on area coverage (Supplementary Table 2). All 26 articles were focused and recruited participants in a suitable way and as such the appraisal was continued. The second series of questions focused on how bias was eliminated in the research. Scores of 14.5/26 were satisfactory. Confounding factors were either not declared or not often mentioned within the participant recruitment trial method. In terms of diabetes, there were no studies where a large proportion of participants were diabetic, which otherwise could result in effecting the CAUTI outcome. Studies were either balanced in terms of the number of males and female in each catheter group or the entire study only had participants of a single sex which makes the studies suitable for inclusion in the meta-analysis. Diabetes mellitus if undiagnosed or not controlled is considered a risk factor due to potential presence of sugars in the urine, which can promote both bacterial colonisation and multiplication to a greater extent than in urine with a lower sugar concentration [64, 65]. Some articles only monitored for UTI incidence for a few days whilst some monitored for a longer period. From this all the research was found to be of moderate quality and above; therefore, none of the articles were subsequently removed from the identified studies for this review.

### Meta analysis

**Fig. 2:**
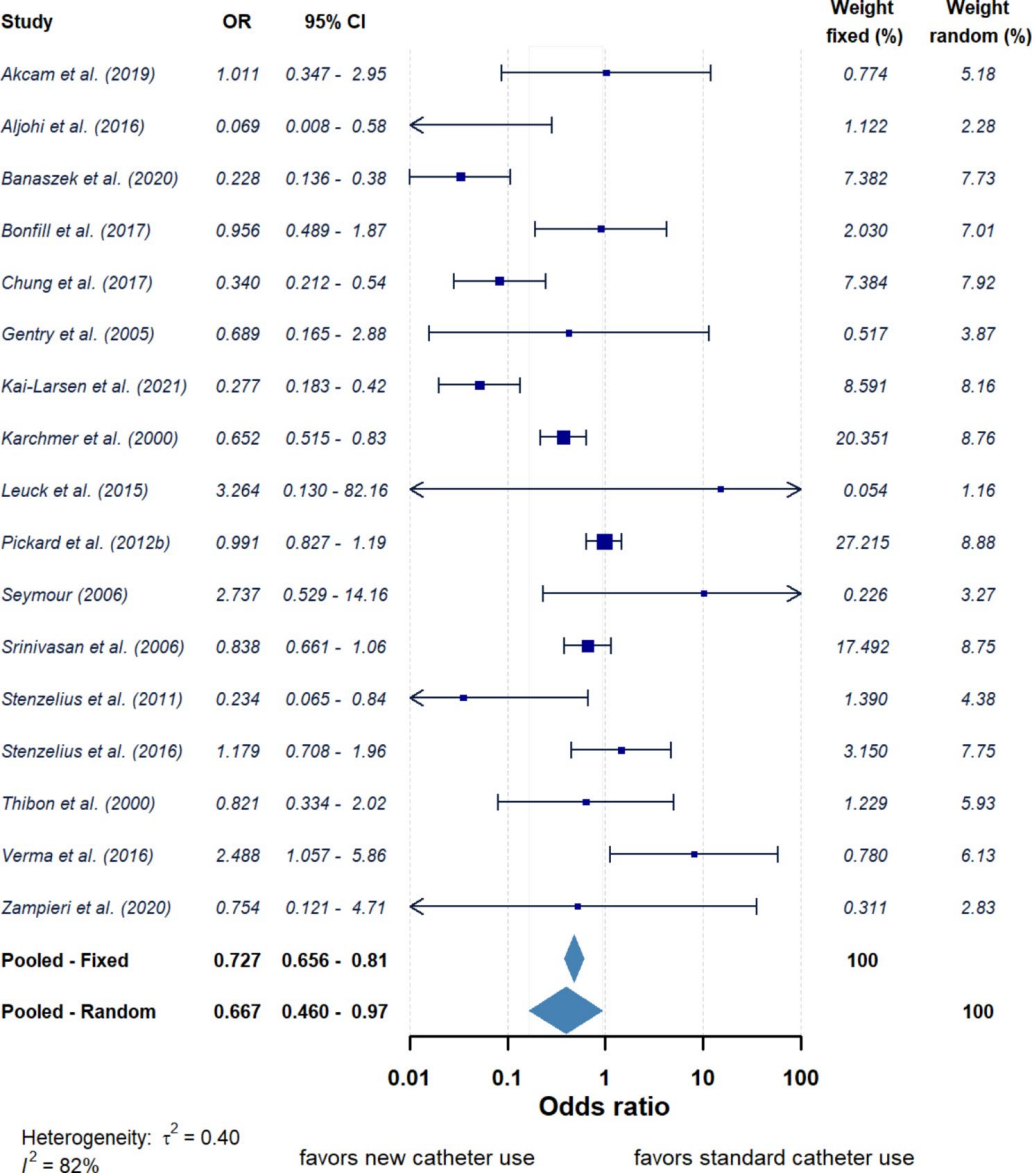
Forest plot of risk (reporting odds ratio (OR) and 95% confidence interval (CI)) of developing UTI between patients using new silver-based antimicrobial catheters against standard catheters

**Fig. 3:**
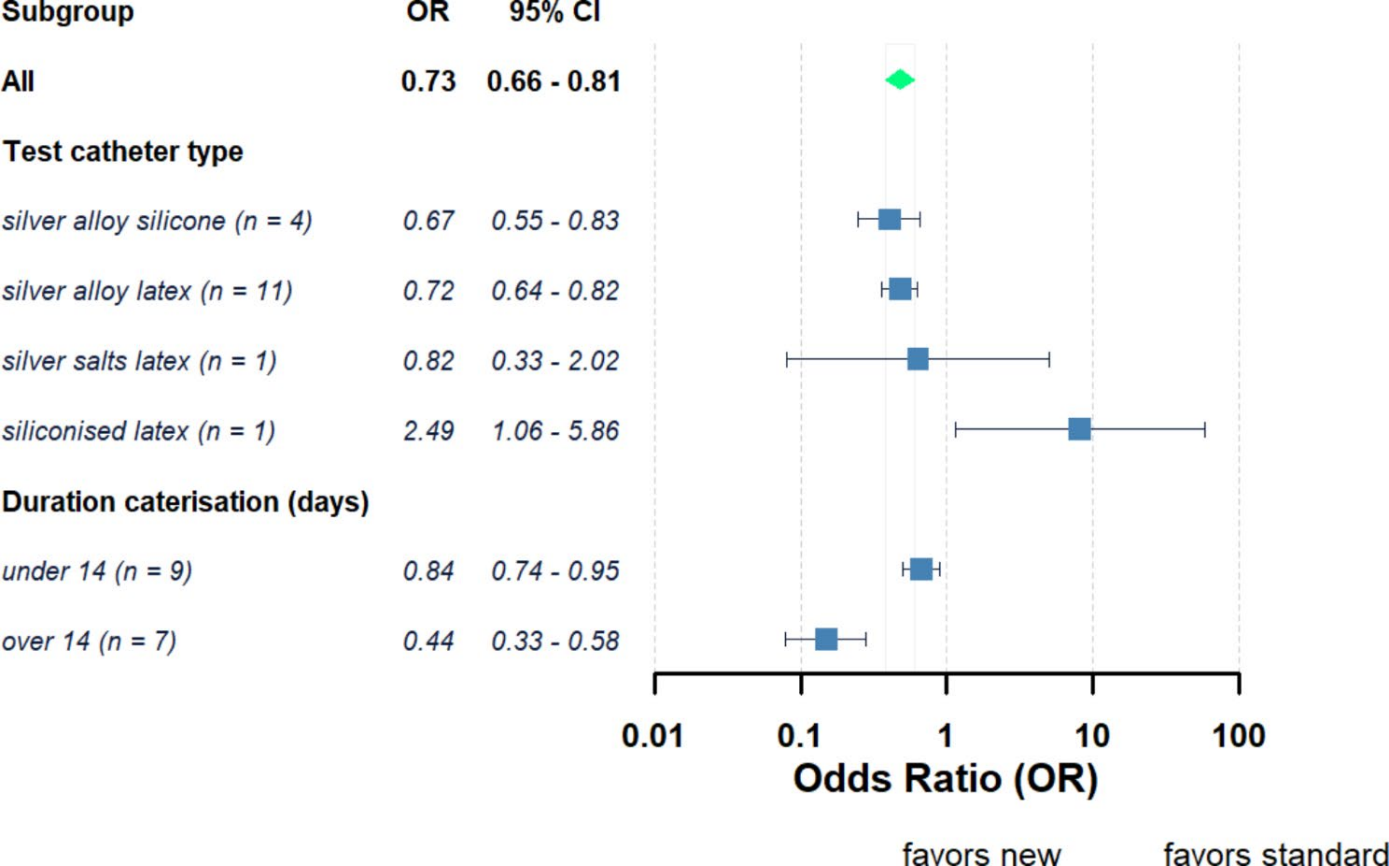
Pooled odds ratios (ORs) and 95% confidence interval (CIs) of developing UTI between patients using new silver-based antimicrobial catheters against standard catheters grouped according to new silver-based catheter material characteristics

Across those studies using silver-based antimicrobial catheters compared with standard catheters resulted in a range of ORs between 0.07 and 3.26 (Fig. 2). The pooled OR of the 17 studies which used silver-based catheters was 0.73 (95% CI = 0.66–0.81, *P* < 0.05) and 0.67 (95% CI = 0.46–0.97, *P* < 0.05) for fixed and random effects models, respectively. Overall, this suggests use of silver-based catheters reduce the odds of developing a UTI compared to using a standard catheter. However, the calculated τ² and *I*² values were 0.40 and 82% suggesting dissimilarity amongst these articles. When the silver-based catheters were grouped according to catheter material (Fig. 3) the OR for silver-alloy latex catheters (*n* = 11) was 0.72 (95% CI = 0.64–0.82, *P* < 0.05) for fixed model and 0.60 (95% CI = 0.40–0.92, *P* < 0.05) for random model (Supplementary Fig. 1b). The OR for grouped silver-alloy silicone catheters (*n* = 4) was 0.67 (95% CI = 0.55–0.83, *P* < 0.05) for fixed model and 0.62 (95% CI = 0.25–1.54, *P* > 0.05) for random model (Supplementary Fig. 1a). This suggests silver-alloy nanoparticles embedded in standard catheter materials reduced the UTI odds compared to those standard materials. Moreover, the longer the duration of the catheterisation the greater the reduction of the UTI using silver-based materials with OR for pooled studies with catheterisation < 14 days 0.84 (95% CI = 0.74–0.95) against an OR for studies with duration of catheterisation ≥ 14 days of 0.44 (95% CI = 0.33–0.58).

**Fig. 4:**
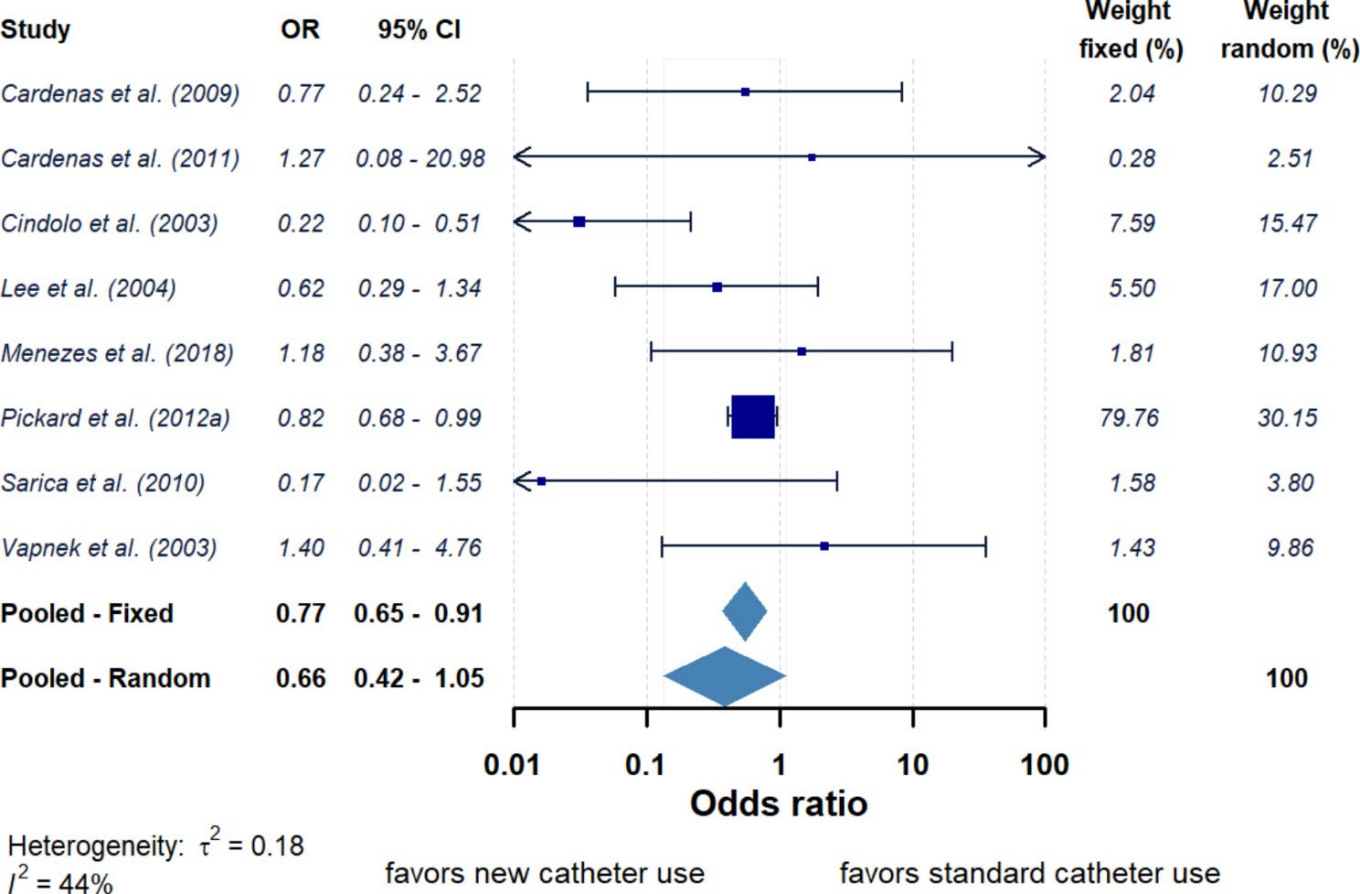
Forest plot of risk (reporting odds ratio (OR) and 95% confidence interval (CI)) of developing UTI between patients using new non-silver-based antimicrobial catheters against standard catheters

**Fig. 5:**
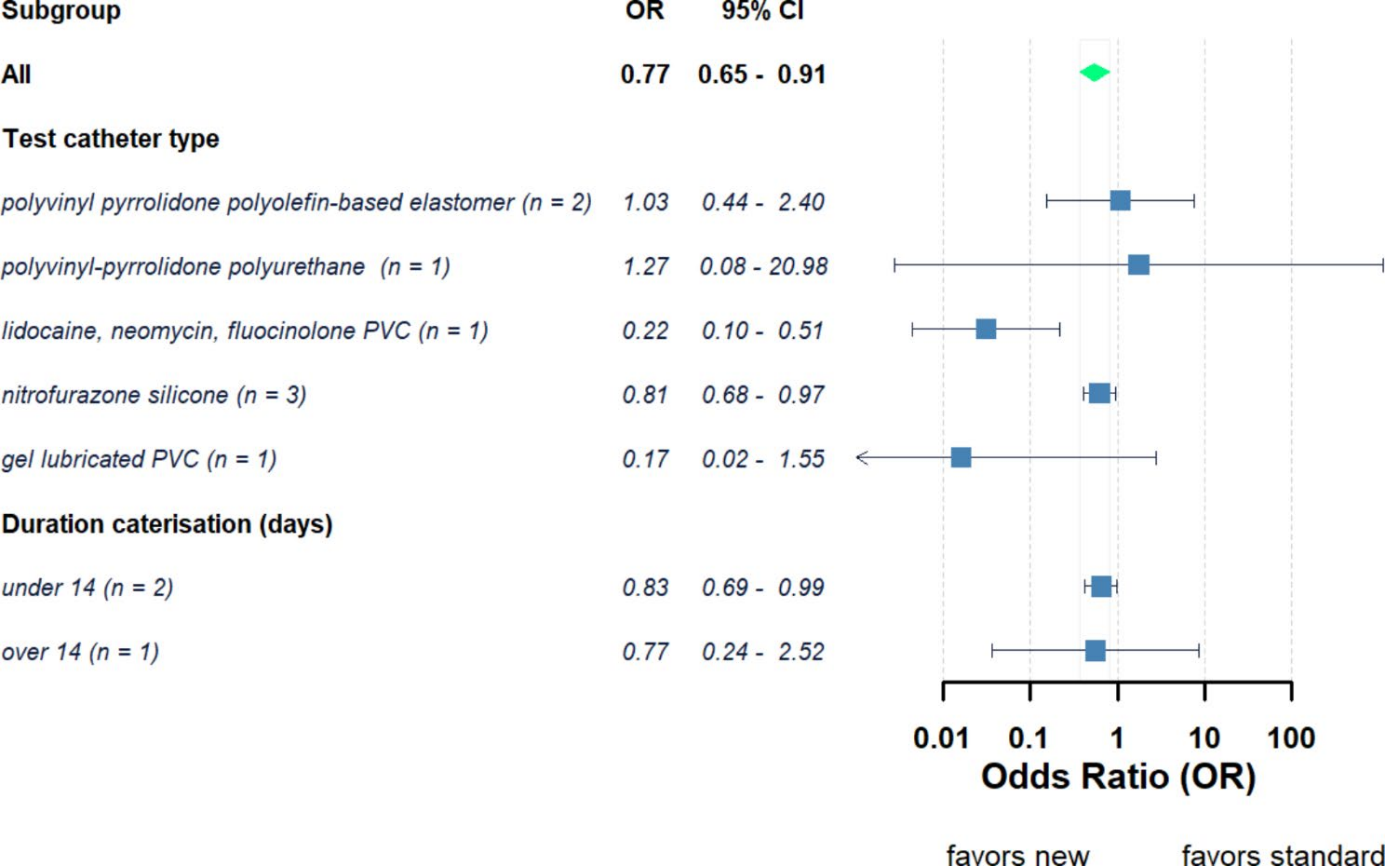
Pooled odds ratios (ORs) and 95% confidence interval (CIs) of developing UTI between patients using new non-silver-based antimicrobial catheters against standard catheters grouped according to new antimicrobial catheter material characteristics

Across those studies using non-silver-based antimicrobial catheters compared with standard catheters resulted in a range of ORs between 0.17 and 1.40 (Fig. 4). The pooled OR for the 8 studies which used non-silver-based catheters was 0.77 (95% CI = 0.65–0.91, *P* < 0.05) and 0.66 (95% CI = 0.42–1.05, *P* > 0.05) for fixed and random effects models respectively. Overall, this suggests use of non-silver-based catheters reduce the odds of developing a UTI compared to when using a standard catheter. However, the calculated τ² and *I*² values were 0.18 and 44% suggesting greater similarity amongst these articles than those studies which used silver-based catheters. When the non-silver-based catheters were grouped based on catheter material (Fig. 5) the OR for the nitrofurazone silicone catheters (*n* = 3) was 0.81 (95% CI = 0.68–0.97, *P* < 0.05) for fixed model and 0.81 (95% CI = 0.68–0.97, *P* < 0.05) for random model (Supplementary Fig. 2a). The OR for polyvinyl-pyrrolidone polyolefin-based elastomer catheters (*n* = 2) was 1.03 (95% CI = 0.44–2.40, *P* > 0.05) for fixed model and 1.03 (95% CI = 0.44–2.41, *P* > 0.05) for random model (Supplementary Fig. 2b). No difference was observed instead when studies were grouped based on the duration of the catheterisation (< 14 or ≥ 14 days).

### UTI causative species

**Fig. 6:**
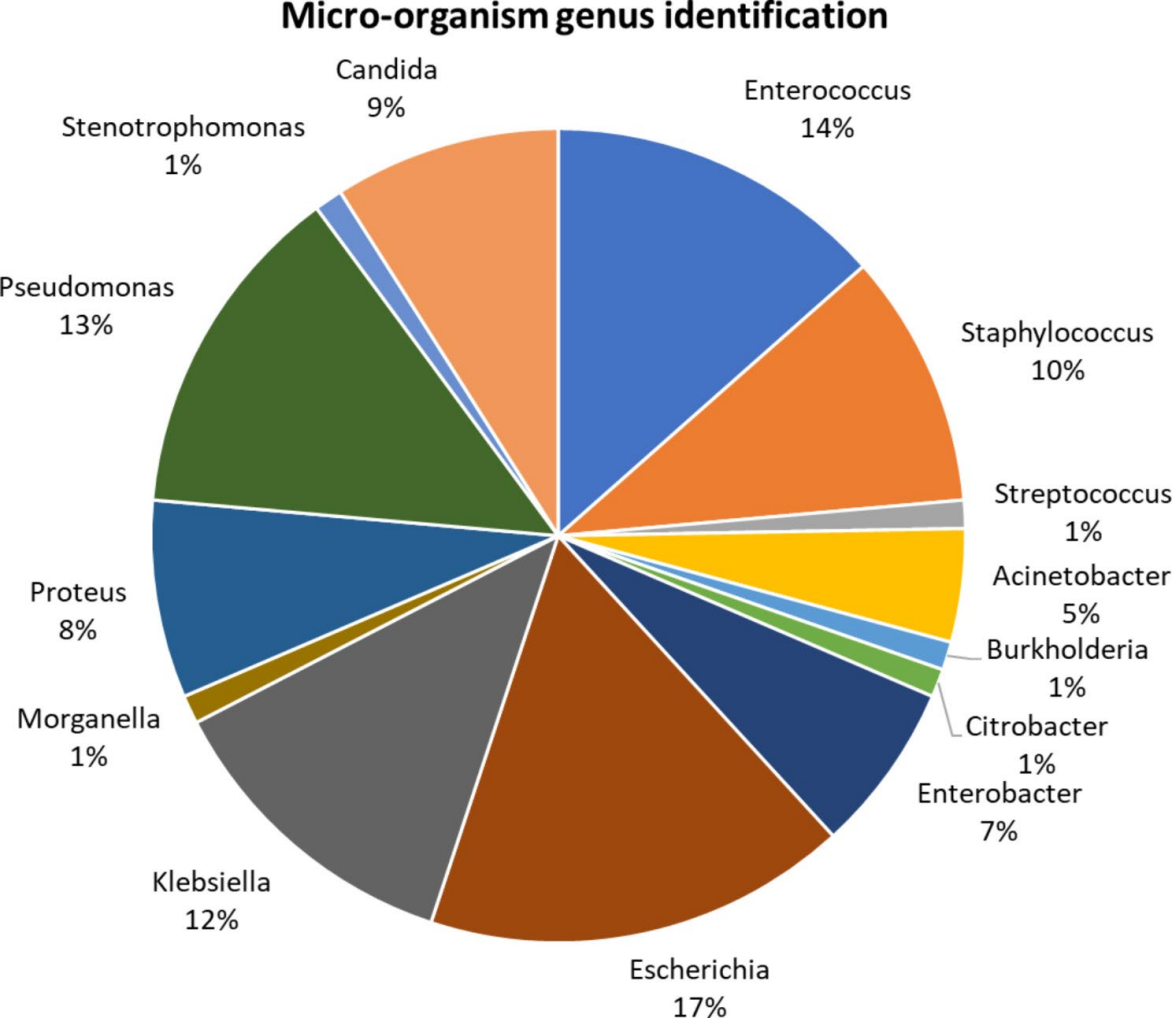
Micro-organism genus involved in CAUTI identified in included studies

Most articles (*n* = 16) identified micro-organisms causative of the UTIs at the genus level (Fig. 6) with some articles identifying to the species level (Supplementary Table 2). Reports differed with some identifying only genus and some confirming at the species level (Supplementary Table 3). Species identified in multiple articles were *Escherichia coli* (*n* = 15), *Klebsiella pneumonia* (*n* = 7) and *Enterococcus faecalis* (*n* = 7). Genus identification in multiple articles included *Pseudomonas spp.* (*n* = 12) and *Enterococcus spp.* (*n* = 12). Bacteria which were only identified in single articles was *Enterococcus faecium*, *Enterococcus gallinarum*, *Staphylococcus epidermidis*, *Streptococcus agalactiae*, *Burkholderia cepacia*, *Acinetobacter baumanni/haemolyticus*, *Morganella morganii*, *Proteus vulgaris*, *Pseudomonas fluorescens* and *Stenotrophomonas maltophilia*. Only half articles identified *Candida spp.* yeast, with species identified *Candida albicans* (*n* = 4), *Candida kefyr* (*n* = 1), *Candida parapsilosis* (*n* = 1) and *Candida tropicalis* (*n* = 1).

## Discussion

A previous systematic review of catheter materials carried out by Beattie and Taylor (2011) [66] included clinical trials and other reviews results and concluded that silver-alloy catheters reduce likelihood of developing UTI when compared with silicone or latex catheters. Their study combined both clinical trials (*n* = 5) reported at time of publishing (2011) and, dissimilar to this review, other systematic reviews (*n* = 6) which had carried out meta-analyses. Only one of their selected clinical trials was included in the present review, Thibon et al. (2000) [60] as all others predated 2000. As Beattie and Taylor review was published in 2011, the present review has added a further 10 years of clinical trials comparing further development in catheters materials. Moreover, Beattie and Taylor only included silver-alloy catheters as test catheters which compared to the present review has both benefits and drawbacks. Whilst their study has greater focus on test catheter it lacked the scope of available catheter devices which have been trialled in clinical settings in the years after the review was conducted. Finally, like with the present review, Beattie and Taylor identified significant heterogeneity, which limits conclusions that can be made. Another recent review and meta-analysis also addressed the role of catheter materials on CAUTI but the focus was only on PVC material for intermittent catheterisation [67]; therefore covering a narrower range of materials then this work.

Here it was found that silver-alloy latex and nitrofurazone silicone catheters had a UTI incidence with OR < 1 (*P* < 0.05) indicating suitability for reducing risk of developing CAUTI. In contrast, silver-alloy silicone and polyvinyl-pyrrolidone polyolefin-based elastomer catheters returned odds ratios and 95% CI range which suggests no statistically significant difference in UTI incidence compared to standard catheters. The dissimilarity in catheter comparison is perhaps highlighted in the calculated τ² and *I*² values, which are indicative of a dissimilar comparison being made amongst the articles; silver-alloy silicone (τ² = 0.33 and *I*² = 83%), silver-alloy latex (τ² = 0.57 and *I*² = 86%) suggesting considerable heterogeneity. The non-silver-based catheter grouping was calculated as having moderate heterogeneity (τ² = 0.18 and *I*² = 44%), despite being a group of various catheters compared against standard catheters.

Risk for CAUTIs is not only dependent on the catheter material, but is mainly related to other factors surrounding the care and use of them; this could explain both some of the heterogeneity between studies and the reason that an effect from catheter material may only be a small part of the overall CAUTI risk. The remaining heterogenicity could be attributed to the large variability in the studies identified, such as, length of catheterisation and limited number of studies representing each catheter type which matched the desired criteria for meta-analysis [68–70]. Heterogeneity could be reduced in future studies if more defined search criteria focused on a single test catheter material; however, this could also have considerable detrimental impact if only a few studies are identified matching the research criteria. If the pool of catheters assessed was smaller than patient comfort and hospital costs and patient length of stay could be considered to greater extent. At present it appears that silver-alloy based catheters would be the most suitable devices for use in clinical environments if further research is not to be carried out.

Although nitrofurazone catheters were calculated with an OR favouring their use, fluoroquinolones such as nitrofurazone are now being avoided as they are a potential carcinogen [71]. Other catheter devices when grouped returned similar OR to silver-alloy silicone suggesting perhaps catheter assignment should be based on patient infection risk in specific healthcare institutions. Given the large number of studies with 95% CI values encompassing the OR of 1 there is a need for new catheter devices to be developed which enable a significant reduction in the risk of a patient developing CAUTI. Therefore, any future catheter devices which are developed should be tested for antibacterial effect, including bacterial anti adherence properties, and compared with silver-alloy ion releasing materials.

The duration of the catheterisation is known to impact the probability of CAUTI, short-term catheterisation has been attributed to have a 5% increase in the daily infection rate [18] whilst longer durations can increase that up to 10% per day [72]. We carried out sub-group analysis pooling studies with long (over 14 days) and short (under 14 days) catheterisation and observed that the impact of silver-based material appeared to improve with longer catheterisation instead of waning as suggested instead by Maki and Tambyah et al. (2001) [18]; this could be attributed to the fact that despite the silver release from the catheter surface the material still was able to exhibit antimicrobial activity. This was observed instead of the non-silver based materials, however the number of studies in this cases was very small and such uncertainty is a major point in any inference. Another area worth revisiting would be the infection definition used as asymptomatic bacteriuria is not often considered of grouped as part of CAUTI. Therefore, more specific outcomes could be investigated as it would be more crucial to identify and prevent instances of bacteriuria which often have symptoms which cause patient discomfort. As with diabetes considered here, other confounding factors which could be considered in future studies include, age, sex, UTI recurrence [73], and SCI given a recent systematic literature review convincingly demonstrating that SCI patients are at higher risk of UTI, not because of indwelling catheterisation carrying a greater UTI risk but rather because SCI patients being immunodeficient [21]. As such, future studies should either only utilise SCI patients using indwelling catheters as the cohort or adjust the outcomes for bias if only a proportion of patients are SCI patients.

The presence of *E. coli* as the micro-organism most frequently found across the studies correlates with the consensus that this bacterium is most often responsible for UTIs. Furthermore *Klebsiella pneumonia*,* Proteus mirabilis*, *Pseudomonas aeruginosa* and *Staphylococcus aureus* have been previously identified as UTI causative, as found by the articles in this review [24, 74]. *Staphylococcus saprophyticus*, a species not wholly identified in any of the 16 micro-organism identifying articles has also been identified in previous work [75]. Identification of coagulase-negative *Staphylococcus* (identified in 6 studies), which includes the identified *S. epidermidis* could indeed include *Staphylococcus saprophyticus*, which as a member of coagulase-negative Staphylococcus [76]. Given *Staphylococcus saprophyticus* prevalence in causing UTI [77], as reported in other literature, this could be an ideal target along with the micro-organisms identified with both genus and species name. *Candida spp.* identification also correlates with the literature with *Candida spp.* being the most common fungal nosocomial causative agent for UTI, more specifically the greatest prevalence of *Candida albicans* [78], in agreement with the findings of this research. Candiduria is most often seen in patients as asymptomatic UTIs [79], which would only be identified by bacterial counting if the patient presented no symptoms. From the literature it is suggested that asymptomatic candiduria does not require administration of antifungal drugs, such as fluconazole, as there are no benefits to patients clinically [80, 81]. 

Overall, the selected research here correlates with the general literature in terms of the UTI causative micro-organisms and as such, *E. coli*, *K. pneumonia*, *P. mirabilis*, coagulase-negative *Staphylococcus*, *E. faecalis*, *P. aeruginosa* and *C. albican*s should all be considered when trialling newly designed catheters.

Catheter material research suggests that surface roughness and material hydrophobicity play a role in bacterial adherence and subsequent biofilm formation. Coated latex was found to be rougher with increased biofilm formation compared to smoother silicone with lesser biofilm formation [82], whilst *P. mirabilis* biofilm formation was greater on rougher siliconised latex compared to silicone [83]. Hydrophobicity of catheter surface plays a role in bacterial adherence and subsequent biofilm formation with latex having a more hydrophobic surface than silicone [84], with hydrophobic bacteria adhering to surfaces with greater ease than to those which are hydrophilic [85, 86]. The UTI incidence comparing silver-alloy latex and silicone is not clear from the odds ratio calculated, with similar OR values of 0.72 and 0.67 respectively. Out of the three non-trademarked materials, siliconised latex, PVC and polyurethane, the greatest observed adherence was that of *S. aureus* to PVC [87]. In this research siliconised latex was found to have an OR of 2.49 indicating a high UTI incidence. Given the UTI incidence of siliconised latex and *S. aureus* adherence to PVC, standard materials should be modified and surface roughness considered if new devices are to be developed.

This review provides a contemporary insight into what modifications have been carried out to the standard materials that could inspire research in new materials and modifications. The list of micro-organisms collected could provide not only a revision on the UTI causative micro-organisms for healthcare considerations but also for research opportunities providing rationale for the selection of the micro-organisms to be employed in testing of new catheter materials under development. Further strengths of this review is the large number of modern trials identified and forming part of the meta-analysis. However, given the mixed comparisons gathered in the present systematic review, with the high dissimilarity calculated in meta-analysis, it is difficult to provide an overall conclusion to what would be the most suitable catheter for hospital use. Furthermore, the statistical outcomes regarding heterogeneity suggest the studies are perhaps too dissimilar to be compared, even when separated into smaller groups which consist of the same test catheter.

## Conclusions

Our review has suggested that silver-alloy based catheters would be the preferred catheter type as this material can significantly reduce the incidence of UTI’s in patients requiring catheterisation. However, latex should be avoided due to the potential for patient allergies. Ultimately, this highlights the need and warrants further research to help identify and develop a catheter device material that will help eradicate the incidence for UTIs.

Ultimately, it is anticipated that by identifying a catheter type which may significantly reduce the clinical incidences of UTIs, it could allow current NHS protocols to be revised, improved and implemented within 5 years, for those patients requiring catheterisation as part of their treatment.

### Electronic supplementary material

Below is the link to the electronic supplementary material.

Supplementary Material 1

## Data Availability

All data generated or analysed during this study are included in this published article and its supplementary information files.
