# Supplementary material for: Materials-based incidence of urinary catheter associated urinary tract infections and the causative micro-organisms: systematic review and meta-analysis
Source: BMC Urol. 2024 Aug 30;24:186. doi: 10.1186/s12894-024-01565-x (PMC11363627; doi:10.1186/s12894-024-01565-x)
Supplement: Supplementary file 1 — Supplementary Material 1 [file 12894_2024_1565_MOESM1_ESM.docx]

Supplementary information

**Supplementary Table 1.** String search employed to identify potentially relevant research articles, (A): Ovid MEDLINE, (B): PubMed and (C): Web of Science

(A)

| # | Search | Results |
| --- | --- | --- |
| 1 | exp Urinary Catheter/ or urinary catheter.mp | 3,789 |
| 2 | commercial catheter.mp | 23 |
| 3 | foley catheter | 2,679 |
| 4 | indwelling catheter.mp | 2,387 |
| 5 | #1 or #2 or #3 or #4 | 8,544 |
| 6 | exp Urinary Tract Infection/ or urinary tract infection.mp | 62,192 |
| 7 | urinary catheter associated infection.mp | 2 |
| 8 | #6 or #7 | 62,192 |
| 9 | catheter material.mp | 232 |
| 10 | catheter coating.mp | 39 |
| 11 | encapsulated catheter.mp | 1 |
| 12 | #9 or #10 or #11 | 271 |
| 13 | #5 and #8 and #12 | 18 |

(B)

| # | Search | Results |
| --- | --- | --- |
| 1 | urinary catheterization[MeSH Terms] | 14,845 |
| 2 | urinary catheter[All Fields] | 18,806 |
| 3 | commercial catheter[All Fields] | 2,255 |
| 4 | foley catheter [All Fields] | 3,566 |
| 5 | indwelling catheter[All Fields] | 26,500 |
| 6 | ((((indwelling catheter[All Fields]) OR (foley catheter[All Fields])) OR (commercial catheter[All Fields])) OR (urinary catheter[All Fields])) OR (urinary catheterization[MeSH Terms]) | 51,576 |
| 7 | urinary tract infection[MeSH Terms] | 49,266 |
| 8 | urinary tract infection[All Fields] | 76,294 |
| 9 | urinary catheter associated infection[All Fields] | 4,038 |
| 10 | ((urinary catheter associated infection[All Fields]) OR (urinary tract infection[All Fields])) OR (urinary tract infection[MeSH Terms]) | 76,920 |
| 11 | catheter material[All Fields] | 17,065 |
| 12 | catheter coating[All Fields] | 2,869 |
| 13 | encapsulated catheter[All Fields] | 324 |
| 14 | ((encapsulated catheter[All Fields]) OR (catheter coating[All Fields])) OR (catheter material[All Fields]) | 18,593 |
| 15 | ((((encapsulated catheter[All Fields]) OR (catheter coating[All Fields])) OR (catheter material[All Fields])) AND (((urinary catheter associated infection[All Fields]) OR (urinary tract infection[All Fields])) OR (urinary tract infection[MeSH Terms]))) AND (((((indwelling catheter[All Fields]) OR (foley catheter[All Fields])) OR (commercial catheter[All Fields])) OR (urinary catheter[All Fields])) OR (urinary catheterization[MeSH Terms])) | 1,001 |

(C)

| # | Search* | Results |
| --- | --- | --- |
| 1 | TOPIC:(urinary tract infection) OR TOPIC:(urinary catheter associated infection) | 50,838 |
| 2 | TOPIC:(urinary catheter) OR TOPIC:(commercial catheter) OR TOPIC:(foley catheter) OR TOPIC:(indwelling catheter) | 20,488 |
| 3 | TOPIC:(catheter material) OR TOPIC:(catheter coating) OR TOPIC:(encapsulated catheter) | 13,950 |
| 4 | #1 AND #2 AND #3 | 1,011 |

*= Indexes = SCI-EXPANDED, SSCI, A&HCI, CPCI-S, CPCI-SSH, ESCI Timespan = All years

**Supplementary Table 2.** Report of Critical Appraisal Skills Programme (CASP) outcome. Responses to questions, yes = ✓, not sure = ? , no = 🗶. For CASP score yes = 1, not sure = 0.5, no = 0 points

| Article | Did the study address a clearly focused issue? | Was the cohort recruited in an acceptable way? | Was the exposure accurately measured to minimise bias? | Was the outcome accurately measured to minimise bias? | Have the authors identified all important confounding factors? | Have they taken account of the confounding factors in the design and/or analysis? | Was the follow up of subjects complete enough? | Was the follow up of subjects long enough? | Do you believe the results? | Can the results be applied to the local population? | Do the results of this study fit with other available evidence? | CASP score |
| --- | --- | --- | --- | --- | --- | --- | --- | --- | --- | --- | --- | --- |
| Akcam et al. (2019) | ✓ | ✓ | ✓ | ✓ | ? | ✓ | ✓ | ✓ | ✓ | ✓ | ✓ | 10.5 |
| Aljohi et al. (2016) | ✓ | ✓ | ✓ | ✓ | 🗶 | ✓ | ✓ | 🗶 | ✓ | ✓ | ✓ | 9 |
| Banaszek et al. (2020) | ✓ | ✓ | ? | ? | 🗶 | 🗶 | ? | ? | ? | ✓ | ✓ | 6.5 |
| Bonfill et al. (2017) | ✓ | ✓ | ✓ | ? | ✓ | ✓ | ✓ | ✓ | ✓ | ✓ | ✓ | 10.5 |
| Cardenas et al. (2009) | ✓ | ✓ | ? | ✓ | 🗶 | 🗶 | ✓ | ✓ | ✓ | ✓ | ? | 8 |
| Cardenas et al. (2011) | ✓ | ✓ | 🗶 | ✓ | ? | ? | ✓ | ✓ | ✓ | ✓ | ? | 8.5 |
| Chung et al. (2017) | ✓ | ✓ | ? | ✓ | ✓ | ✓ | ? | ✓ | ✓ | ✓ | ✓ | 10 |
| Cindolo et al. (2003) | ✓ | ✓ | ✓ | ✓ | ✓ | ✓ | ✓ | ✓ | ✓ | ✓ | ✓ | 11 |
| Gentry et al. (2005) | ✓ | ✓ | ? | ✓ | ? | 🗶 | ✓ | ✓ | ? | ✓ | ✓ | 8.5 |
| Kai-Larsen et al. (2021) | ✓ | ✓ | ✓ | ✓ | ✓ | ? | ✓ | 🗶 | ✓ | ✓ | ✓ | 9.5 |
| Karchmer et al. (2000) | ✓ | ✓ | ? | ✓ | 🗶 | 🗶 | ✓ | ✓ | ✓ | ✓ | ✓ | 8.5 |
| Lederer et al. (2014) | ✓ | ✓ | ? | ✓ | 🗶 | 🗶 | ✓ | ✓ | ✓ | ✓ | ✓ | 8.5 |
| Lee et al. (2004) | ✓ | ✓ | ? | ✓ | ✓ | ✓ | ✓ | ✓ | ✓ | ✓ | ? | 10 |
| Leuck et al. (2015) | ✓ | ✓ | 🗶 | ✓ | ✓ | ? | ✓ | ✓ | ✓ | ✓ | ? | 9 |
| Magnusson et al. (2019) | ✓ | ✓ | 🗶 | ✓ | ? | ? | ✓ | ✓ | ✓ | ? | ✓ | 8.5 |
| Menezes et al. (2018) | ✓ | ✓ | 🗶 | ✓ | ✓ | ✓ | ✓ | ✓ | ✓ | ✓ | ? | 9.5 |
| Pickard et al. (2012) | ✓ | ✓ | ? | ✓ | 🗶 | 🗶 | ✓ | 🗶 | ✓ | ✓ | ✓ | 7.5 |
| Sarcia et al. (2010) | ✓ | ✓ | ✓ | ✓ | 🗶 | 🗶 | ✓ | ✓ | ? | ✓ |  | 8 |
| Seymour (2006) | ✓ | ✓ | ? | ✓ | 🗶 | 🗶 | ✓ | ✓ | ✓ | ✓ | ✓ | 8.5 |
| Srinivasan et al. (2006) | ✓ | ✓ | ? | ✓ | 🗶 | 🗶 | ✓ | ✓ | ✓ | ✓ | ✓ | 8.5 |
| Stenzelius et al. (2011) | ✓ | ✓ | ? | ✓ | ✓ | ✓ | ✓ | ✓ | ✓ | ✓ | ✓ | 10.5 |
| Stenzelius et al. (2016) | ✓ | ✓ | 🗶 | ? | 🗶 | 🗶 | ✓ | ✓ | ✓ | ✓ | ✓ | 7.5 |
| Thibon et al. (2000) | ✓ | ✓ | ✓ | ✓ | 🗶 | 🗶 | ✓ | ✓ | ✓ | ✓ | ✓ | 9 |
| Vapnek et al. (2003) | ✓ | ✓ | ? | ✓ | 🗶 | 🗶 | ✓ | ✓ | ✓ | ✓ | ? | 8 |
| Verma et al. (2016) | ✓ | ✓ | ? | ✓ | 🗶 | 🗶 | ✓ | ? | ✓ | ✓ | ? | 7.5 |
| Zampieri et al. (2020) | ✓ | ✓ | ✓ | ✓ | ✓ | 🗶 | ✓ | ✓ | ✓ | ✓ | ✓ | 10 |

**Supplementary Table 3.** Micro-organism identification collected across the research cohort at both genus and species level


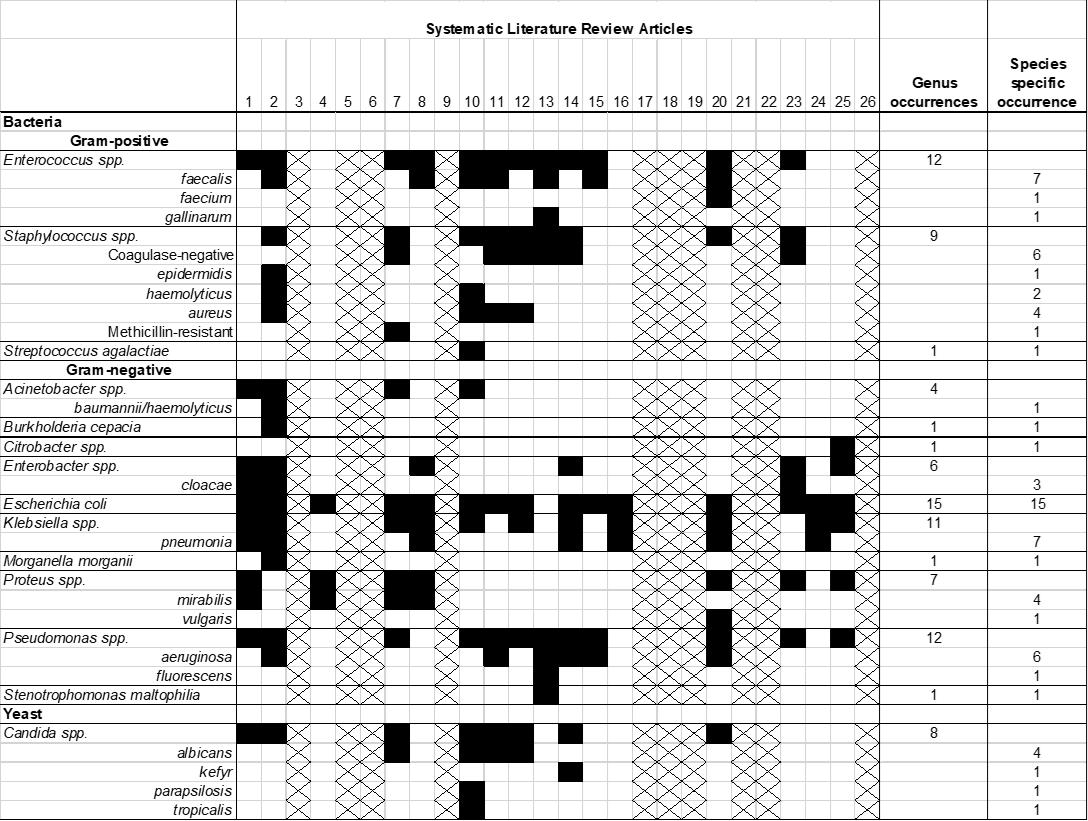


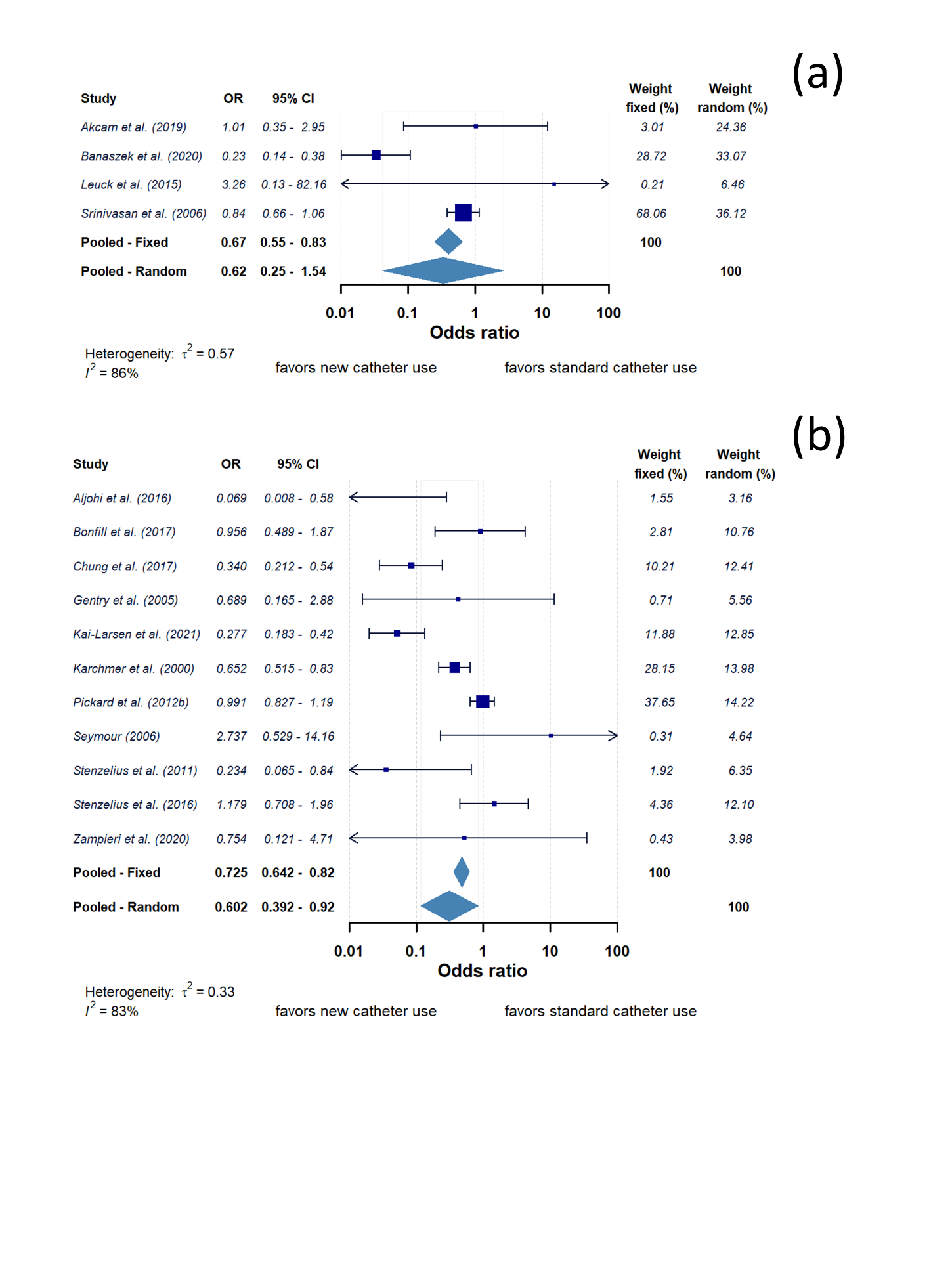
**Supplementary Figure 1**. Forest plot of risk (reporting odds ratio (OR) and 95% confidence interval (CI)) of developing UTI between patients using new (**a**) silver alloy silicone and (**b**) silver alloy latex catheters against standard catheters.


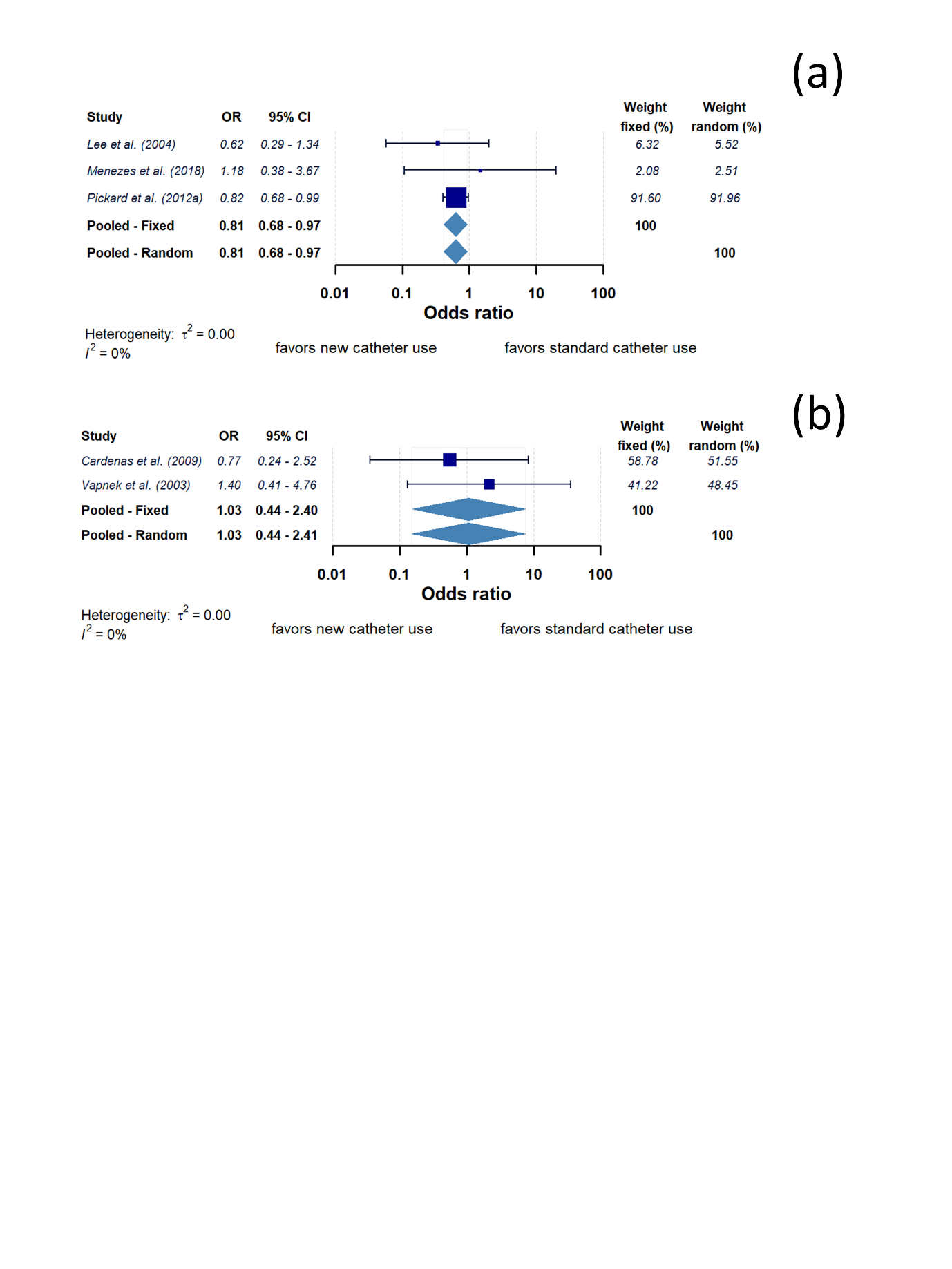


**Supplementary Figure 2**. Forest plot of risk (reporting odds ratio (OR) and 95% confidence interval (CI)) of developing UTI between patients using new nitrofurazone silicone (**a**) and polyvinyl pyrrolidone polyolefin-based elastomer and (**b**) catheters against standard catheters.


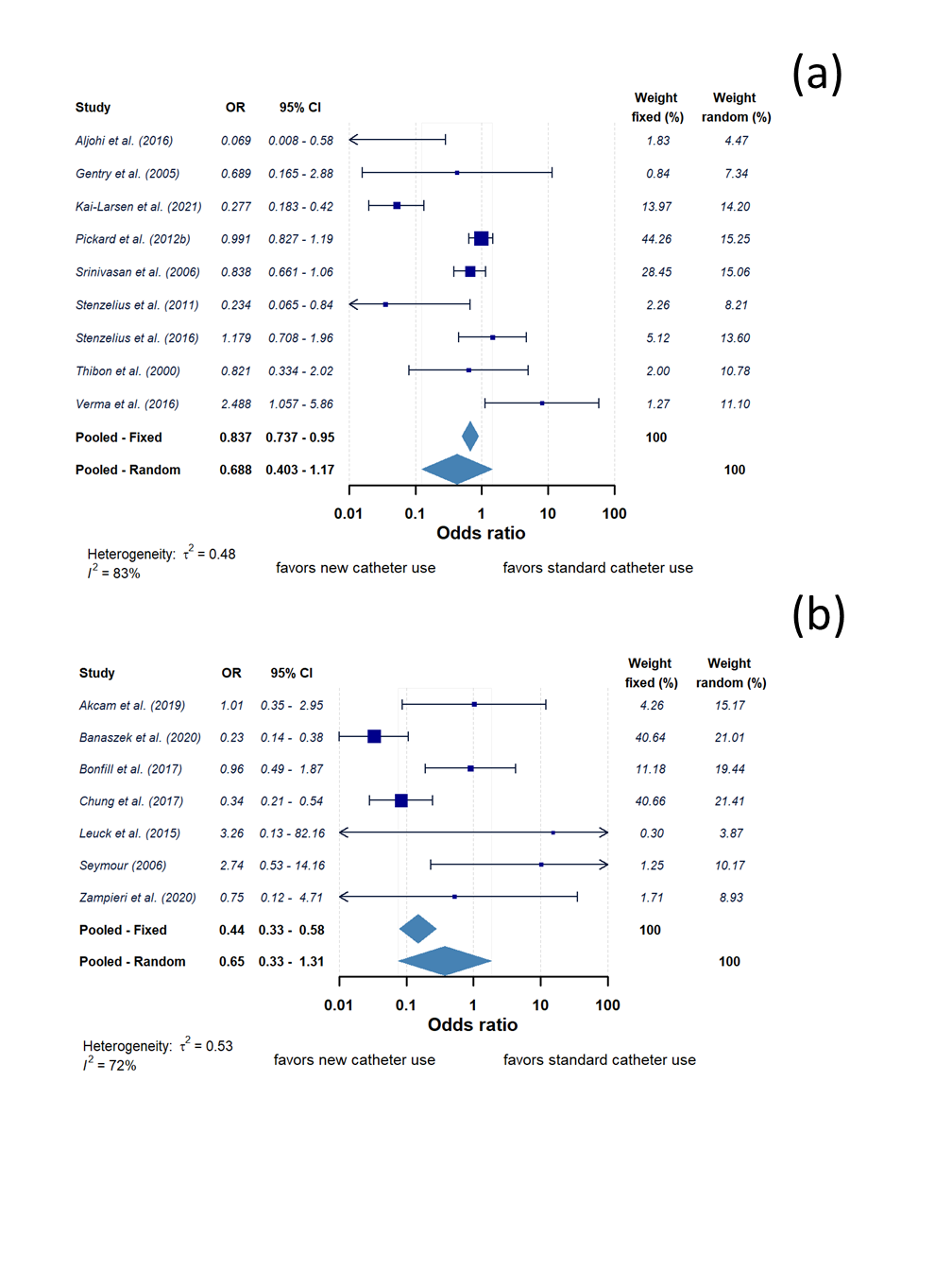


**Supplementary Figure 3**. Forest plot of risk (reporting odds ratio (OR) and 95% confidence interval (CI)) of developing UTI between patients using new silver-based catheters for (**a**) short duration (<14 days) (**b**) long duration (≥14 days) against standard catheters.


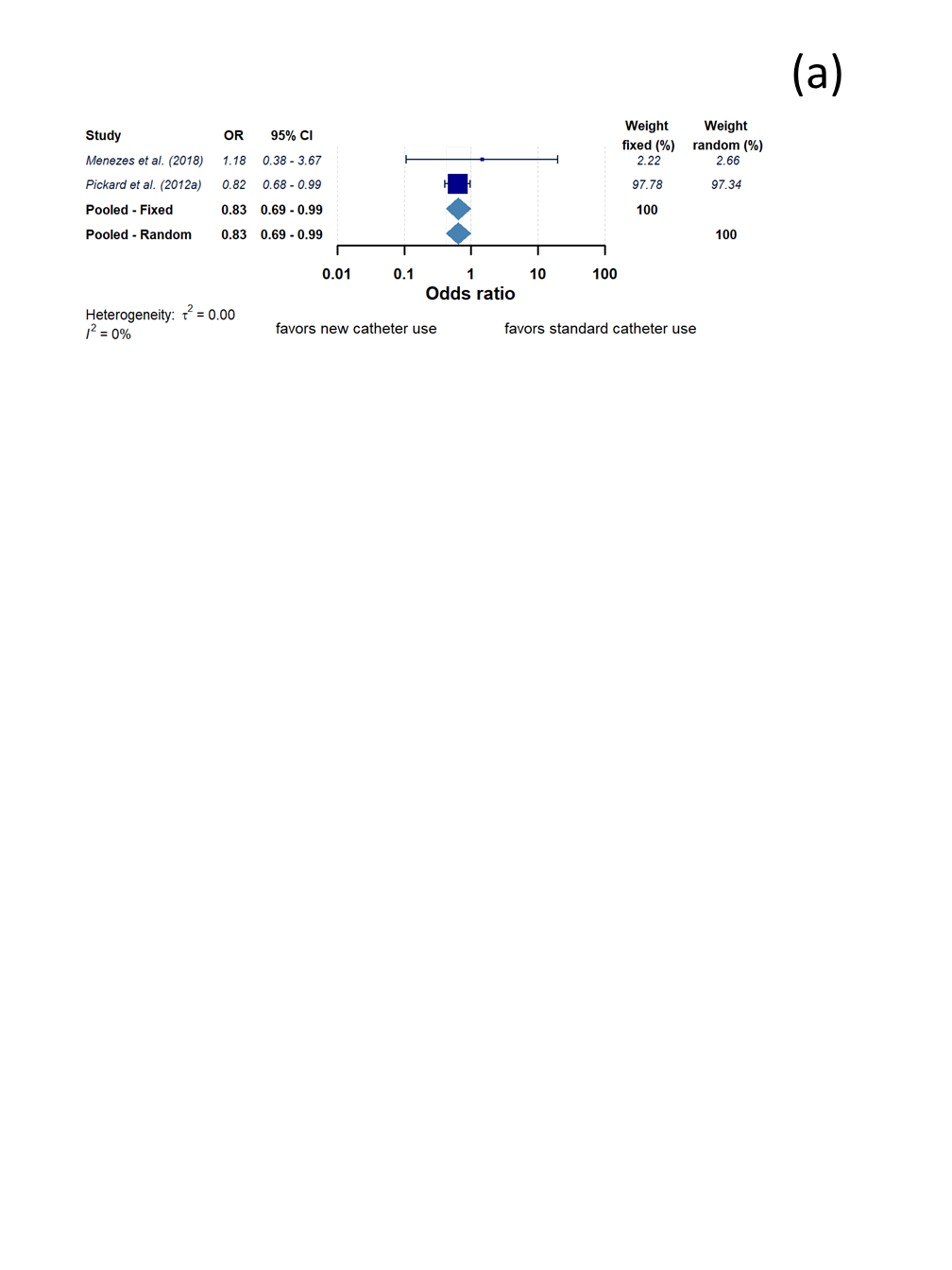


**Supplementary Figure 4**. Forest plot of risk (reporting odds ratio (OR) and 95% confidence interval (CI)) of developing UTI between patients using non-silver-based antimicrobial catheters for short duration (<14 days) against standard catheters.
